# Seasonal shifts in the assembly dynamics of benthic macroinvertebrate and diatom communities in a subtropical river

**DOI:** 10.1002/ece3.5904

**Published:** 2020-01-03

**Authors:** Jun Wang, Jiancheng Hu, Tao Tang, Jani Heino, Xiaoming Jiang, Zhengfei Li, Zhicai Xie

**Affiliations:** ^1^ State Key Laboratory of Freshwater Ecology and Biotechnology Institute of Hydrobiology Chinese Academy of Sciences Wuhan China; ^2^ University of Chinese Academy of Sciences Beijing China; ^3^ Freshwater Centre Finnish Environment Institute Oulu Finland

**Keywords:** Chishui River, functional diversity, functional traits, lotic assemblages, null model

## Abstract

Identifying seasonal shifts in community assembly for multiple biological groups is important to help enhance our understanding of their ecological dynamics. However, such knowledge on lotic assemblages is still limited. In this study, we used biological traits and functional diversity indices in association with null model analyses to detect seasonal shifts in the community assembly mechanisms of lotic macroinvertebrates and diatoms in an unregulated subtropical river in China. We found that functional composition and functional diversity (FRic, FEve, FDis, MNN, and SDNN) showed seasonal variation for macroinvertebrate and diatom assemblages. Null models suggested that environmental filtering, competitive exclusion, and neutral process were all important community assembly mechanisms for both biological groups. However, environmental filtering had a stronger effect on spring macroinvertebrate assemblages than autumn assemblages, but the effect on diatom assemblages was the same in both seasons. Moreover, macroinvertebrate and diatom assemblages were shaped by different environmental factors. Macroinvertebrates were filtered mainly by substrate types, velocity, and COD_Mn_, while diatoms were mainly shaped by altitude, substrate types, and water quality. Therefore, our study showed (a) that different biological assemblages in a river system presented similarities and differences in community assembly mechanisms, (b) that multiple processes play important roles in maintaining benthic community structure, and (c) that these patterns and underlying mechanisms are seasonally variable. Thus, we highlight the importance of exploring the community assembly mechanisms of multiple biological groups, especially in different seasons, as this is crucial to improve the understanding of river community changes and their responses to environmental degradation.

## Graphical abstract

(a) Null models suggested that environmental filtering, competitive exclusion, and neutral process were all important community assembly mechanisms for both biological groups. (b) Different biological assemblages in a river system presented similarities and differences in assembly mechanisms. (c) Patterns and underlying mechanisms are seasonally variable.

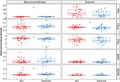

## 1 INTRODUCTION

Current theoretical and empirical frameworks emphasize that biological communities are structured by a combination of deterministic and stochastic processes (Csercsa et al., 2019; Devercelli, Scarabotti, Mayora, Schneider, & Giri, 2016; Patrick & Swan, 2011; Swan & Brown, 2011). The deterministic perspective assumes that the distributions of species are under environmental filtering (i.e., local environmental conditions) and biotic interactions (e.g., Chase & Myers, 2011; Giam et al., 2016; Heino, Gröroos, Soininen, Virtanen, & Muotka, 2012; Isabwe et al., 2018), while the stochastic perspective assumes that species distributions are mainly determined by random births, deaths, and dispersal assembly (Grönroos et al., 2013; Hubbell, 2001; Tonkin et al., 2018). Under the framework of stochastic and deterministic processes, both taxonomic and functional measures have been used to disentangle the relative roles of multiple processes in lotic community assembly (e.g., Medina Torres & Higgins, 2016; Sokol, Benfield, Belden, & Valett, 2011). Among these measures, trait‐based measures have the most extensive adoption, due to the fact that traits are sensitive to environmental changes and can provide key insights into ecosystem functioning (e.g., Adler, Fajardo, Kleinhesselink, & Kraft, 2013; Aiba et al., 2013; Mason, Bello, & Mouillot, 2013; Obertegger & Flaim, 2018).

Previous studies on community assembly in freshwater systems commonly focused on specific periods of time (e.g., Heino & Tolonen, 2017; Obertegger & Flaim, 2018). However, streams and rivers are highly heterogeneous ecosystems with significant spatiotemporal variation in community structure (Ward, Tockner, Arscott, & Claret, 2002). For most rivers, hydrology, water temperature, and nutrients change seasonally (Fitzgerald, Winemiller, SabajPerez, & Sousa, 2017; Leung, Liao, & Dudgeon, 2012; Rosemond, Mulholland, & Brawley, 2000). Because of changes in these factors, harsh seasons could strengthen environmental filtering to select the most suitable traits from the regional species pool to occur at a site (Chase, 2007; Hart & Finelli, 1999). For instance, Stenger‐Kovács, Lengyel, Crossetti, Üveges, and Padisák (2013) demonstrated that high‐ and low‐profile (i.e., tall and short stature, respectively; Passy, 2007) diatom species increased with increased solar irradiance, because higher irradiance levels can penetrate the algal matrix and can reach the deeper layers of the epilithon (Dodds, 1992). In addition, macroinvertebrate scrapers and predators reached their highest abundance during summer due to maximal resource levels (Álvarez‐Cabria, Barquín, & Juanes, 2010). Moreover, biotic interactions, such as grazing and predation, are also important in the context of community assembly (Abe, Uchida, Nagumo, & Tanaka, 2006; Bolam, Rollwagen‐Bollens, & Bollens, 2019; Vilmi, Tolonen, Karjalainen, & Heino, 2017), which can lead to temporal variation in lotic community structures (Fitzgerald et al., 2017; Rosemond et al., 2000; Tonkin et al., 2018; Yang, Tang, & Dudgeon, 2009).

Seasonal changes in community assembly mechanisms may also differ among assemblages (Heino et al., 2012; Isabwe et al., 2018). In freshwater systems, benthic macroinvertebrates and diatoms are both important groups in biodiversity monitoring and assessments, and their functional trait compositions can also be influenced by seasonality in biotic and abiotic factors (Bêche, Mcelravy, & Resh, 2006; Stenger‐Kovács et al., 2013). Benthic diatoms often show spatiotemporal patterns that are mainly shaped by water chemical conditions (Passy & Larson, 2011; Rosemond et al., 2000; Tang, Jia, Jiang, & Cai, 2016; Yang et al., 2009). Moreover, diatom communities are often dominated by low‐profile diatoms such as *Cocconeis* or *Cymbella* at high discharge periods (Poff, Voelz, & Ward, 1990). In addition, due to similar demands for nutrients, interspecific competition may also play an important role in the assembly of diatom communities (Hillebrand, 2005). By comparison, benthic macroinvertebrates, the main consumers in lotic ecosystems, comprise diverse species groups with different life cycles, functional roles, and trophic traits (Göthe, Angeler, Sandin, & Rasmussen, 2013). Temporal variation in community patterns is mainly caused by variation in a myriad of species life history features (Johnson, Carreiro, Jin, & Jack, 2012), while species interactions only play a relatively weak role among lotic macroinvertebrates (de Mendoza et al., 2018). Therefore, the community assembly mechanisms of lotic benthic macroinvertebrates and diatoms may be different due to the difference in their responses to seasonal changes in physio‐chemical conditions and intensity of interspecific interactions.

Although seasonal changes in benthic macroinvertebrate and diatom community structures have been well documented, little attention has been given to seasonal shifts in the underlying assembly mechanisms (Bêche et al., 2006; Csercsa et al., 2019; Göthe et al., 2013; Walters, 2011). This is the case that can be evaluated when macroinvertebrates and diatoms are sampled from the same sites. Here, we surveyed benthic macroinvertebrate and diatom assemblages in April and September (representing spring and autumn, respectively) in an unregulated subtropical river in China. We used trait‐based null models to examine environmental filtering, niche differentiation, and neutral process in different seasons for the community assembly of the two taxonomic groups (Heino & Tolonen, 2017; Sarremejane, Mykrä, Bonada, Aroviita, & Muotka, 2017; Ulrich & Gotelli, 2010). If environmental filtering was important, we further examined how local environmental conditions influenced the community assembly process. We aimed to detect whether (a) functional traits for each taxonomic group differ between April and September, and whether (b) community assembly mechanisms show seasonal shifts for each taxonomic group due to temporal fluctuations in environmental conditions. Answering these questions will provide information on whether lotic benthic macroinvertebrates and diatoms show different community assembly mechanisms and whether there is temporal variability in these mechanisms.

## 2 METHODS AND MATERIALS

### 2.1 Study area

The Chishui River (31°25′–32°48′N, 109°10′–110°45′E) is located in the core area of the National Nature Reserve for rare and endemic fishes of the upper Yangtze River. The reserve was established in 2005 to provide a refuge for endemic fishes due to the construction of the Three Gorges Dam (Jiang et al., 2010; Wu, Huang, Han, Xie, & Gao, 2003). Thus, the mainstream of the Chishui River is less polluted and contains rich species and high biodiversity. Besides, the Chishui River is located in a subtropical area and shows significant seasonal variation in river hydrology (Jiang et al., 2010; Jiang, Xiong, & Xie, 2017). Additionally, there are no dams built on the mainstream, providing an ideal place for identifying contributions of natural environmental filtering, biotic interactions, and dispersal limitation to lotic community assembly. The Chishui River covers a total drainage area of 20,440 km^2^ and has a mainstream length of 436.5 km. The annual precipitation is 1,027.2 mm, and approximately 60% of rainfall occurs between June and September (Mao, Guo, Deng, Xie, & Tang, 2018).

### 2.2 Fieldwork and laboratory methods

**Figure 1:**
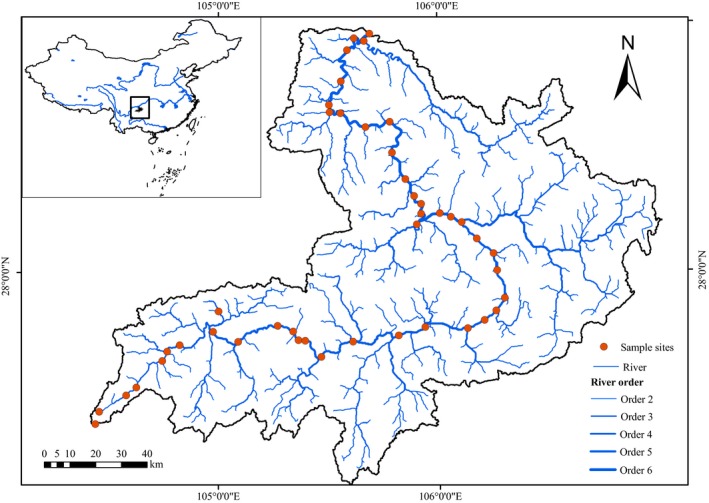
Map showing the 44 sampling sites (red circles) of benthic macroinvertebrates and diatoms collected in the Chishui River. The square box represents the location of the basin in China

Benthic Macroinvertebrates and diatoms were collected from 44 mainstream sites in April and September 2016 (Figure 1). April has relative stable environmental conditions for aquatic organisms, while September is the recovery period after summer floods in the Chishui River (Jiang et al., 2017). At each site, we took three random quantitative samples using a Surber sampler (30 × 30 cm, 500 μm in mesh size) to collect benthic macroinvertebrates on each sampling occasion (Chi et al., 2017; Jiang et al., 2010). Then, macroinvertebrate specimens were sorted on a white plate in the field and were preserved in 70% alcohol. In the laboratory, specimens were identified to genus or lower taxonomic level according to appropriate references (Brinkhurst, 1986; Dudgeon, 1999; Epler, 2001; Morse, Yang, & Tian, 1994; Zhou, Gui, & Zhou, 2003), and the abundance (i.e., number of individuals) was counted for each taxon.

At the same sites where macroinvertebrates were collected, five moveable stones (with a diameter range of 15–60 cm) were randomly selected to collect benthic diatom samples. The sampling area was confined using a circular lid (radius: 2.7 cm). For each stone, the surface within the lid was vigorously scrubbed using a nylon brush and rinsed three to four times with distilled water. All subsamples from each site were combined into one composite sample, and its volume was recorded. The diatom sample was preserved in 4% formalin for further identification and enumeration. In the laboratory, diatoms were identified and enumerated to species level, after the sample was acid‐cleaned and slide‐mounted, using 1,000 × magnification with an oil immersion objective under a compound microscope (Olympus CX21: Olympus Optical Co.) (Hu & Wei, 2006). Diatoms were identified following the taxonomic references of Qi (1995), Shi (2004), Krammer (2000, 2002, 2003), and Lange‐Bertalot, Bak, Witkowski, and Tagliaventi (2001), and relative abundance was calculated for each taxon.

We measured physical habitat and water chemistry variables at each sample site during each sampling occasion. Altitude was recorded with a GPS system (Garmin GPS‐76 system). Current velocity was measured with a current flow meter (FP211; Global Water). Substrate types were classified into four main types (Barbour, Gerritsen, Snyder, & Stribling, 1999): cobble (64–256 mm), pebble (16–64 mm), gravel (2–16 mm), and sand and silt (0.25–2 mm), and measured according to the percentage of each type in the sample section. Conductivity (Cond), pH, and dissolved oxygen (DO) were measured with a portable Yellow Springs Instrument (YSI) meter (Model 33; YSI, Incorporated, Yellow Springs). Meanwhile, Additional 600 ml stream water sample was collected and preserved in acidic conditions to measure total nitrogen (TN), total phosphorus (TP), ammonium nitrogen (${\text{NH}}_{4}^{+}-N$), and permanganate index (COD_Mn_) in the laboratory based on the standard methods recommended by the national water monitoring protocol (Wei, 2002). TN and TP concentrations were determined using a combined persulfate digestion followed by spectrophotometric analysis. ${\text{NH}}_{4}^{+}-N$ concentrations were measured by the indophenol blue method (Apha, 1995; Wei, 2002), and COD_Mn_ was measured by titration with acidic potassium permanganate (Wei, 2002).

### 2.3 Biological traits and functional diversity

Thirteen traits for macroinvertebrates and 14 traits for diatoms were selected to represent three trait categories characterizing body sizes, habitat associations, and functional groups (Table 1). These traits play important roles in community assembly processes for the two biological groups (Merritt & Cummins, 1996; Stenger‐Kovács et al., 2013; Tolonen, Hämäläinen, Holopainen, Mikkonen, & Karjalainen, 2003). The body size determines key behavioral and life history characteristics such as growth rates and metabolism (Passy, 2007), as well as interactions between individuals within and between species (Pawar, 2015). The habitat associations indicate living habitat for macroinvertebrates and how algal taxa associate with substrate, reflecting habitat preference of lotic organisms. Functional feeding groups characterize resource use, food, and feeding behavior (Heino & Tolonen, 2017). All trait information was assigned to genus level following literature for each taxonomic group (Brinkhurst, 1986; Jiang et al., 2010; Law, Elliott, & Thackeray, 2014; Liu, Zhang, Wang, & Wang, 1979; Morse et al., 1994; Poff et al., 2006; Rimet & Bouchez, 2012; Stenger‐Kovács et al., 2013). Genus level has been proved to be adequate to preserve the biotic community information in previous studies (Poff et al., 2006; Rimet & Bouchez, 2012). We used a fuzzy coding procedure (Chevenet, DolÉ Dec, & Chessel, 1994; Usseglio‐Polatera, Bournaud, Richoux, & Tachet, 2000) to describe the trait category for each taxon with a score ranging from 1 indicating “low affinity” to 5 indicating “high affinity” to each trait.

**Table 1:** Functional traits and trait modalities used in this study selected for benthic macroinvertebrate and diatom taxa

Category	Macroinvertebrates		Diatoms	
	Traits	Code	Traits	Code
Body size	Small (<9 mm)	Size1	Size < 100 μm^3^	Size1
	Medium (9–16 mm)	Size2	100 ≤ Size < 300 μm^3^	Size2
	Large (>16 mm)	Size3	300 ≤ Size < 600 μm^3^	Size3
			600 ≤ Size < 1,500 μm^3^	Size4
			Size ≥ 1,500 μm^3^	Size5
Habitat associations	Burrowers	FFG1	Prostrate	P
	Crawler	FFG2	Erect	E
	Semisessiles	FFG3	Stalked	S
	Sessile	FFG4	Filamentous	F
	Swimmers	FFG5	Prostrate and mobile	P + M
			Unattached	U
Functional groups	Gather–collecting	Habit1	Low‐profile	Low
	Filtering–collecting	Habit2	High‐profile	High
	Scraper	Habit3	Motile	Mot
	Shredders	Habit4		
	Predators	Habit5		

We used the community‐weighted mean (CWM) trait value, a trait‐level index, to summarize the distribution of values within each trait for each measured assemblage (Garnier et al., 2004). The index is calculated as the sum across all species of the products of each species trait value and their relative abundance in a given assemblage (Garnier et al., 2004):

$$\text{CWM}=\sum_{i=1}^{n}p_{i}\times trait_{i}$$

where *p_i_* is the relative abundance of *i*th species in the community, *trait_i_* is the trait values of species *i*, and *n* is the total number of species. CWM has been proved to be useful for summarizing shifts in mean trait values within communities due to environmental selection for certain functional traits (Ricotta & Moretti, 2011).

Five community‐level functional diversity metrics were also calculated to examine the community patterns (Fitzgerald et al., 2017). Functional richness (FRic) was measured as the convex hull and estimates overall niche volume, representing proportion of functional space filled by a community. Functional dispersion (FDis) was calculated as the mean distance to centroid and estimates position relative to the center of niche space, representing the dispersion of species in trait space (Laliberté & Legendre, 2010). Functional evenness (FEve) was measured as evenness of branches of a minimum spanning tree, representing the regularity of abundance distributions in the functional space (Mason, Mouillot, Lee, & Wilson, 2005; Mouchet, Villeger, Mason, & Mouillot, 2010; Villeger, Mason, & Mouillot, 2008). Mean nearest neighbor (MNN) distance and standard deviation of nearest neighbor (SDNN) distance estimate how close and even species are in functional trait space, respectively. These two metrics focus on distances between species, with the assumption that biotic interactions lead to more variable trait distribution (Aiba et al., 2013; Webb, Ackerly, McPeek, & Donoghue, 2002).

### 2.4 Statistical analysis

We first explored seasonal differences in functional composition and diversity for each taxonomic group by comparing the observed CWM for each trait and functional diversity metric between April and September. Then, we detected season‐specific assembly processes for each taxonomic group. We calculated the standardized effect size (SES) for each functional diversity metric of local assemblages (FRic, FEve, FDis, MNND, and SDNN). SES was calculated as (observed value–mean simulated value)/*SD* of simulated (Gotelli & McCabe, 2002), in which the observed value was measured functional diversity and the simulated values were inferred from a null model using the matrix‐swap algorithm with 1,000 random runs. SES values higher and lower than 0 indicate overdispersion and underdispersion, respectively (Swenson & Enquist, 2009). Overdispersion suggests actions of competition, limiting similarity or character displacement, whereas underdispersion indicates operations of abiotic or biotic filtering (de Bello et al., 2012). Furthermore, to explore environmental driving forces the assembly process, generalized linear model with stepwise selection was conducted to select key environmental variables. This analysis was only performed if a functional diversity metric showed significant underdispersion. Finally, by comparing the above metrics of the two biological groups between April and September separately, we determined whether community assembly processes differed between benthic macroinvertebrates and diatoms.

Functional diversity metrics were calculated using the “dbFD” function in the R package “FD” (Laliberté, Legendre, & Shipley, 2014). Seasonal differences in functional diversity metrics for each taxonomic group were examined using the Mann–Whitney *U* test. Significant dispersion from random assembly was assessed using a two‐sided Wilcoxon rank sum test. The null model used the matrix‐swap algorithm that was implemented via the function “RandomizeMatrix” in the R package “picante” (Kembel et al., 2010). The generalized linear model with stepwise selection was calculated using the function “glm” and “step” in the R package “stats.”

## 3 RESULTS

A total of 224 macroinvertebrate and 183 diatom taxa were observed in the present study, with 191 and 146 for macroinvertebrates and 120 and 107 for diatoms in April and September, respectively. Dominant macroinvertebrate taxa were *Stictochironomus devinctus* (with a mean relative abundance of 13.13%)*, Chironomus* sp. (6.12%), *Cinygmina* sp. (6.00%), *Baetis* sp. (5.43%) in April, and *Baetis* sp. (9.67%), *Cinygmina* sp (7.43%), *Heptagenia* sp. (6.83%), *Cheumatopsyche* sp. (6.61%), *Baetiella* sp. (5.04%) in September. For diatoms, *Achnanthes minutissima* (33.4%), *Cocconeis placentula* (19.3%), *Gomphonema parvulum* (14.3%), *Achnanthes linearis* (11.4%) were dominant in April, with *A. minutissima* (46.0%), *C. placentula* (13.7%), and *G. parvulum* (5.5%) dominating in September.

**Figure 2:**
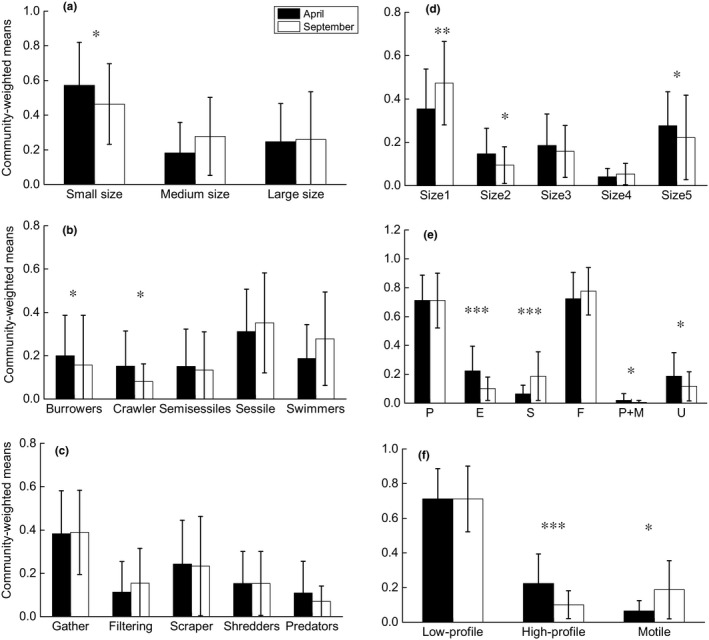
Comparisons of community‐weighted means (CWMs) of functional traits for (a), (b), and (c) macroinvertebrates and (d), (e), and (f) diatoms between April and September based on the Mann–Whitney *U* test. *p < .05, **p < .01, ***p < .001

**Figure 3:**
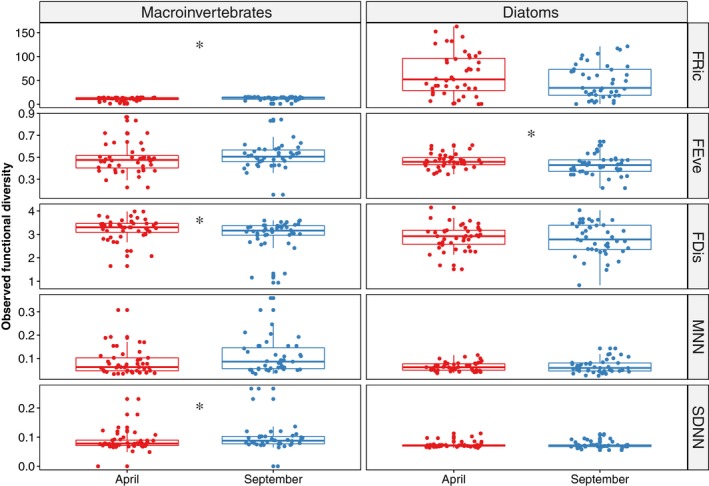
Comparisons of observed functional diversity metrics of local macroinvertebrate and diatom assemblages during April and September. Functional diversity metrics refer to mean nearest neighbor distance (MNN), standard deviation of nearest neighbor distance (SDNN), functional richness (FRic), functional evenness (FEve), and functional dispersion (FDis). Test statistics refer to Wilcoxon rank sum test. *Significant difference (*p* < .05)

Several biological traits and functional diversitry metrics displayed significant seasonal differences for the both taxonomic groups (Figure 2). CWMs of macroinvertebrates with small body size (*W* = 1,247.5, *p* = .020), burrowers (*W* = 1,260.5, *p* = .015), and crawlers (*W* = 1,271, *p* = .012) decreased in September when compared with those in April. As for diatoms, CWMs of size1 class (*W* = 604, *p* = .005), stalked taxa (*W* = 442, *p* < .001), and motile diatoms significantly increased (*W* = 1,185, *p* = .023) in September. In contrast, CWMs of size2 class (*W* = 1,213, *p* = .013), size5 class (*W* = 1,200, *p* = .017), erect (*W* = 1,382, *p* < .001), P + M (*W* = 1,182, *p* = .024), and unattached taxa (*W* = 1,189, *p* = .022) decreased. Macroinvertebrate FRic (*W* = 638, *p* = .014) and SDNN (*W* = 699.5, *p* = .025) were significantly higher, and FDis (*W* = 1,219, *p* = .037) was significantly lower in September than in April (Figure 3). For diatoms, only FEve (*W* = 1,174, *p* = .032) was significantly lower in September than in April. Observed MNN for the two biological assemblages showed nonsignificant differences between the two seasons (Figure 3).

By comparing the null models, we found seasonal differences in community assembly processes and different relations with environmental forces for the two taxonomic groups. Macroinvertebrates in April showed significant underdispersion based on FRic, MNN, and SDNN, but most of the SES values (4 out of 5) in September showed no significant difference from zero (Table 2). Macroinvertebrate functional diversity metrics were mostly related to substrate, velocity, and COD_Mn_ in April (Table 3). Diatom assemblages showed significant underdispersion based on FRic and FDis in April and FDis, MNN, SDNN in September. On the contrary, FEve in April and FRic in September showed significant overdispersion (Table 2). The functional diversity metrics for diatoms in April were related to substrate, velocity, TN, TP, and conductivity, while they were more strongly related to altitude, substrate, ${\text{NH}}_{4}^{+}-N$, and pH in September (Table 3).

**Table 2:** Results of two‐sided Wilcoxon signed rank test of standard effect sizes (SES) based on the two null modeling approaches for the functional diversity metrics

	Macroinvertebrates			Diatoms		
	Median	*V*	*p*	Median	*V*	*p*
April						
FRic	**−1.121**	**3**	**<.001**	**−0.083**	**39**	**<.001**
FEve	**−0.389**	**309**	**.029**	**0.781**	**1,213**	**.012**
FDis	0.126	499	.963	**−0.298**	**0**	**<.001**
MNN	**−0.471**	**266**	**.008**	−0.036	903	.856
SDNN	−0.288	448	.583	−0.116	774	.196
September						
FRic	0.169	418	.491	**0.841**	**34**	**<.001**
FEve	−0.016	361	.686	0.570	1,126	.083
FDis	0.062	440	.521	**−0.284**	**9**	**<.001**
MNN	0.104	392	.229	**−0.353**	**523**	**<.001**
SDNN	0.225	589	.273	**−0.279**	**507**	**<.001**

Note

Median SES, test statistic (*V*), and *p* value are given. Significant results are presented in bold font. Negative/positive SES values represent underdispersion/overdispersion of trait distribution compared to the random expectation

**Table 3:** Generalized linear model outputs for the functional diversity metrics of benthic macroinvertebrates and diatoms in April and September, respectively. This analysis was only performed for functional diversity metrics that showed significant underdispersion in the null model analysis and only important predictors (*p* < .05) are shown

			April					September			
			Estimates	*SE*	*T* value	*p*		Estimates	*SE*	*T* value	*p*
Macroinvertebrates	FRic	Intercept	14.136	0.834	17.064	<.001	—				
		Substrate	−2.856	0.768	−3.728	<.001					
		Velocity	−2.752	0.682	−4.053	<.001					
	FEve	Intercept	14.323	1.376	10.369	<.001	—				
		COD_Mn_	−1.234	0.512	−2.36	.021					
	MNN	Intercept	11.243	0.493	22.813	<.001	—				
		Substrate	−2.579	0.899	−2.882	.006					
Diatoms	FRic	Intercept	24.224	29.354	0.825	.414	—				
		Substrate	32.076	10.093	3.179	.003					
		TN	−20.87	8.544	−2.453	.019					
		TP	592.591	286.192	2.068	.045					
		Cond	0.093	0.036	2.105	.042					
		Velocity	28.572	8.287	3.452	.001					
	FDis	Intercept	2.713	0.143	18.771	<.001	Intercept	−7.582	3.254	−2.334	.025
		Altitude	−0.0005	0.0001	−3.489	.001	pH	1.302	0.392	3.332	.002
		Velocity	0.428	0.103	4.159	<.001	Altitude	−0.001	0.003	−4.781	<.001
	MNN	—					Intercept	0.065	0.012	8.703	<.001
							${\text{NH}}_{4}^{+}-N$	0.103	0.043	2.292	.027
	SDNN	—					Intercept	0.064	0.003	21.651	<.001
							Substrate	0.009	0.003	2.689	.010
							Altitude	0.001	0.001	4.271	<.001

## 4 DISCUSSION

In this study, we used a trait‐based approach to unravel seasonal community assembly dynamics of benthic macroinvertebrates and diatoms. Seasonal changes in CWMs and comparisons of functional diversity metrics with null models for the two taxonomic groups indicated that environmental filtering on functional traits occurred in association with seasonal dynamics (Fortunel, Paine, Fine, Kraft, & Baraloto, 2013). Additionally, limiting similarity and stochastic process also had important effects on assembly dynamics.

Shifts in trait CWMs indicated that environment filtering played important roles in driving seasonal shifts in community assembly through trait selections (Isabwe et al., 2018; Passy & Larson, 2011; Ricotta & Moretti, 2011). Small body‐sized macroinvertebrates sharply decreased in September may due to the high flows in summer leading to elimination of some organisms. In addition, as high flows bring more food resources from the land to aquatic system, organisms no longer need “burrow” and “crawl” habits to avoid stranding and to find food. For diatoms, increase in small cell taxa and motile guild and decrease in larger cell ones and high‐profile guild in September may attribute to high flow condition in summer. Only taxa with small size and relatively strong attachment to the substrate can withstand high flow disturbance (Passy, 2007). Diatom trait CWMs might also been selected by nutrients (Stenger‐Kovács et al., 2013); for instance, high‐profile taxa were dominant in high‐nutrient conditions (such as TP in April; Table S1).

Differences in functional diversity metrics provide further evidence on seasonal changes in community assembly processes. Macroinvertebrate functional diversity showed significant seasonal variation based on FRic, FDis, and SDNN. Functional richness was positively correlated with taxonomic richness (Mayfield et al., 2010; Schmera, Heino, Podani, Erős, & Dolédec, 2017), while functional dispersion was measured based on both richness and abundance (Laliberté & Legendre, 2010). In our study, macroinvertebrate richness and abundance decreased in September, which led to decrease in the convex hull of trait occupation and reduction in trait dispersion. In addition, since the SDNN measure is related to limiting similarity or niche differentiation (Kraft, Valencia, & Ackerly, 2008), the significant increase in SDNN implies that macroinvertebrates showed potential competitive effects in September (such as grazing taxa *Cinygmina* sp. vs. *Heptagenia* sp.). Moreover, higher variation in MNN in September suggests that local assemblages were composed of taxa with complementary functional strategies (Fitzgerald et al., 2017). It was evidenced by seasonal shifts in dominant functional groups, such as *S. devinctus* being replaced by *Cheumatopsyche* sp. and *Baetiella* sp., which showed differences in body size, habitat associations, and functional feeding mechanisms. Compared with the relatively longer life cycles of macroinvertebrates (Young et al., 2014), diatoms generally have shorter life cycles and may thus show more rapid trait‐related changes associated with environmental changes. However, in this study, although there were seasonal changes in the taxonomic and functional composition of diatom communities, *A. minutissima* and *C. placentula* remained invariably abundant (Table S2). The convex hull volume and relative to the center of niche space may largely depend on these two taxa, resulting in nonsignificant changes in functional diversity. However, due to temporal replacement in taxonomic and trait composition and reduction in richness and abundance, functional evenness would significantly decrease from April to September. Additionally, due to the fact that these two taxa both belong to the low‐profile group (Passy, 2007), there might potentially be competition for nutrients among diatoms in both seasons. The probability of competition was also suggested by SES significantly larger than zero for some functional diversity metrics (e.g., FEve) in April and September (e.g., FRic).

Our analysis found that causes of environmental filtering differed substantially between the two biological groups. Environmental filtering played an important role in macroinvertebrate assembly according to the null model analysis. In April, macroinvertebrate communities were largely influenced by substrate types, velocity, and water quality (i. e. COD_Mn_). Local habitat conditions are considered as the main driving factors of macroinvertebrate diversity (Beisel, Usseglio‐Polatera, & Moreteau, 2000; Heino et al., 2012; Johnson, Goedkoop, & Sandin, 2004), and they also proved to be important factors for macroinvertebrate community variation in the Chishui River (Jiang et al., 2017). Relatively smaller habitat space in April, caused by a smaller amount of stream water, had resulted in more limited environmental conditions and stronger environmental filtering effects on biological traits than in September (Horrigan & Baird, 2008). Organic pollution (such as measures as COD_Mn_) was also proved to be an important factor for the distribution of lotic organisms in the Chishui River (Chi et al., 2017; Mao et al., 2018). In addition, suitable current velocity conditions can provide resources (Schoen, Merten, & Wellnitz, 2013) and control sediment compositions (Fenoglio, Boano, Bo, Revelli, & Ridolfi, 2013) for certain species, thereby shaping the composition and abundance of macroinvertebrate communities in the Chishui River (Wang et al., 2018). For benthic diatoms, environmental filtering through physicochemical factors drove community assembly in both seasons. These results are consistent with Mao et al. (2018), who used multiple analytical methods to detect the driving factors that influenced diatom communities in the Chishui River. Altitude and substrate were important for diatoms in both seasons. In the present study, altitude decreased longitudinally from the headwaters to the river's mouth, and was associated with changes in substratum varying from boulder to sand. Hence, diatom communities changed significantly along with these changes (Mao et al., 2018). Furthermore, nutrient supply is also important for diatom species distributions (Passy & Larson, 2011), and thus, traits in diatom communities were more strongly related to water quality variables. However, it is possible that different nutrient variables affect diatoms in different seasons. This may be attributed to increased flows in summer, which could increase the hydrological effects on the stream bed, leading to nutrients being washed from riparian farmland to a water body and resulting in the changes in nutrient concentrations in the river sites in September (Jiang et al., 2017; Mao et al., 2018).

Besides environmental filtering and limiting similarity, we also found that stochastic processes had effects on the community assembly of the two biological groups. Nonsignificant null model results for some metrics (e.g., FDis and SDNN for macroinvertebrates and FEve, MNN, and SDNN for diatoms) are consistent with the idea of stochasticity (Fitzgerald et al., 2017), namely random draws from the regional species pool, random colonization, and drift (Vellend et al., 2014). Moreover, stochastic processes can also be important due to flow disturbances, leading to species extinctions that are decoupled from trait‐based selection (Lepori & Malmqvist, 2009), and due to the random recolonization of some species after avoiding the flood disturbance. Also, previous studies have suggested that stochastic community assembly may have occurred by the equalizing fitness‐related processes and due to trade‐off among traits (Heino & Tolonen, 2017; Mayfield & Levine, 2010; Spasojevic & Suding, 2012). Finally, the simultaneous influence of opposing community assembly mechanisms can also result in random patterns even when the underlying assembly processes are actually highly deterministic (Mouchet et al., 2010; Spasojevic & Suding, 2012; Weiher & Keddy, 1995). Our study focused on the mainstem river. It is likely that seasonal turnover in the species pool may also be influenced by dispersal from tributaries to the mainstem. This effect could bias our results (see also Fitzgerald et al., 2017). However, due to the fact that macroinvertebrates and diatoms were sampled simultaneously on each site, our findings could reveal differences in the strength of assembly mechanisms between these two key biological groups.

## 5 CONCLUSIONS

Our study showed that environmental filtering, biotic interactions, and stochastic processes were all important to community assembly processes of benthic macroinvertebrates and diatoms in the Chishui River. Both physicochemical factors were important to the seasonal shifts of the two taxonomic communities. However, the causes of environmental filtering differed between the biological groups. Therefore, our study highlights the importance of exploring seasonal shifts in assembly dynamics of different biological groups to provide more comprehensive understanding of community assembly mechanisms. Our findings suggest that maintaining diverse substrate types and controlling excess nutrients inputs could be an effective way for sustaining biodiversity in the Chishui River.

## CONFLICT OF INTEREST

None declared.

## AUTHOR CONTRIBUTIONS

T.T. and Z.X. conceived and designed the research; J.W., X.J., Z.L., T.T., and Z.X. performed the fieldwork; J.W. and J.H. analyzed the data; J.W. wrote the manuscript; J.H., T.T., and Z.X. contributed to the discussion; and all authors reviewed the manuscript before submission.

## Data Availability

The datasets analyzed during the current study are available and can be found in Dryad Digital Repository: https://doi.org/10.5061/dryad.h9w0vt4dk
