# Supplementary material for: Seasonal shifts in the assembly dynamics of benthic macroinvertebrate and diatom communities in a subtropical river
Source: Ecol Evol. 2020 Jan 3;10(2):692–704. doi: 10.1002/ece3.5904 (PMC6988552; doi:10.1002/ece3.5904)
Supplement: Supplementary file 1 [file ECE3-10-692-s001.docx]

**Table S1 Environmental physical factors in the Chishui River in April and September (mean ± SD) in 2016.**

|  | April | September |
| --- | --- | --- |
| River orders | 1-6 | 1-6 |
| Altitude (m) | 220-1796 | 220-1796 |
| Boulder (%) | 0.36±0.32 | 0.38±0.33 |
| Cobble (%) | 0.07±0.07 | 0.08±0.11 |
| Pebble (%) | 0.16±0.17 | 0.16±0.17 |
| Sand (%) | 0.39±0.43 | 0.36±0.43 |
| Width (m) | 73.77±59.99 | 52.95±70.33 |
| Velocity (m/s) | 1.02±0.59 | 0.96±0.44 |
| Depth (cm) | 33.32±28.61 | 43.83±53.15 |
| NH4^+-^N (mg/L) | 0.21±0.12 | 0.11±0.10 |
| TN (mg/L) | 3.15±0.91 | 3.64±0.99 |
| TP (mg/L) | 0.05±0.02 | 0.04±0.06 |
| Cond (mg/L) | 478.90±159.95 | 367.54±94.10 |
| DO (mg/L) | 13.20±3.24 | 8.09±0.89 |
| pH | 7.54±1.78 | 8.37±0.20 |
| COD_Mn_ (mg/L) | 2.01±1.04 | 2.43±0.82 |

Note: depth represents where macroinvertebrate and diatom specimen sampled.

**Table S2 Lists of total numbers of taxa, dominant taxa (relative abundance > 5%), and** **taxonomic richness of each functional trait category for macroinvertebrates and diatoms in April and September in 2016.**

|  |  | Macroinvertebrate | |  | Diatoms | |
| --- | --- | --- | --- | --- | --- | --- |
|  |  | April | September |  | April | September |
| Total taxa |  | 191 | 146 |  | 120 | 107 |
| Dominant taxa |  | *Stictochironomus devinctus* (13.13%)*, Cinygmina* sp (6.00 %), *Chironomus* sp. (6.12 %), *Baetis* sp. (5.43 %) | *Baetis* sp. (9.67%), *Cinygmina* sp (7.43%),  *Heptagenia* sp. (6.83%), *Cheumatopsyche* sp. (6.61%), *Baetiella* sp.  (5.04%) |  | *Achnanthes minutissima* (33.4%), *Cocconeis placentula* (19.3%), *Gomphonema parvulum* (14.3%), *Achnanthes linearis* (11.4%) | *Achnanthes minutissima* (46.0%), *Cocconeis placentula* (13.7%), *Gomphonema parvulum* (5.5%) |
| Taxonomic richness of each functional trait category | Burrowers | 38 | 29 | Low-profile | 53 | 44 |
|  | Crawler | 9 | 11 | High-profile | 22 | 24 |
|  | Semisessiles | 51 | 39 | motile | 46 | 39 |
|  | Sessile | 67 | 44 | Size$<$100$\mu m$^3^ | 12 | 8 |
|  | Swimmers | 26 | 24 | 100$\leq$Size$<$300$\mu m$^3^ | 23 | 22 |
|  | Small (< 9 mm) | 82 | 61 | 300$\leq$Size$<$600$\mu m$^3^ | 31 | 23 |
|  | Medium (9–16 mm) | 53 | 33 | 600$\leq$Size$<$1500$\mu m$^3^ | 23 | 28 |
|  | Large (>16 mm) | 56 | 53 | Size$\geq$1500$\mu m$^3^ | 32 | 26 |
|  | Gather-collecting | 46 | 36 | Prostrate | 47 | 33 |
|  | Filtering-collecting | 24 | 19 | Erect | 7 | 9 |
|  | Scraper | 34 | 24 | Stalked | 28 | 28 |
|  | Shredders | 68 | 55 | Filamentous | 3 | 1 |
|  | Predators | 19 | 13 | Prostrate & Mobile | 27 | 29 |
|  |  |  |  | Unattach | 9 | 7 |
